# Fever and Ague

**Published:** 1880-12

**Authors:** 


### FEVER AND AGUE.

Formerly, all medical writers agreed that fever and ague is a disease peculiar to marshy districts, and depending on some subtle poison, called "miasm." No one has ever been able to detect this poison, and its existence is only known by its effect on the system. But, during the past few years, fever and ague has prevailed, where it was supposed that no miasm could exist.

Quinine was once thought to be a specific for all malarial diseases ; but we often see persons suffering from fever and ague in spite of quinine, which they take in large doses daily. Thus two theories in reference to the disease, have been proved to be utterly unreliable. Now there are but few spots in this country where the inhabitants are entirely exempt from malarial diseases; and it seems more than probable that the causes exist more frequently in the individual, his habits and immediate surroundings, than in any poisonous emanations that come to the surface at a *greater* distance. They are improper food, exposure to cold and dampness, and bad ventilation of living and sleeping rooms. These things depress and debilitate the system and poison the blood.

The waste particles of the body are not carried off as fast as they accumulate, and thus the circulation becomes sluggish and the liver torpid and inactive. The wonder is not that this state of things should exist, under these circumstances, but that nature should tolerate them and that recovery should be possible. There may be cases of fever and ague, depending on other diseases ; but all ordinary cases may be completely cured in a week by proper treatment ; relieve the liver of the fearful load under which it is laboring and suffering, and the disease at once ceases. Ten to twenty grains of calomel, taken on going to bed, is our remedy. In a practice of twenty years we have never known this plan to fail. One case in twenty may require a second dose in about a week, but there will be no return of the disease, unless the same causes that produced it are again allowed to act. Dr. Hall's "Old Time" Liver Pills accomplish the same object, and as they are not liable to salivate, many prefer to use them. Fever and ague is liable to favor the development of other diseases, and should therefore be cured as promptly as possible ; and when cured the greatest care should be observed to avoid contracting it again ; therefore avoid over-fatigue, improper food, damp and impure air ; wear flannels next to the skin, and keep the feet dry and warm, and the bowels regular.

